# Design and Characterization of Buccoadhesive Liquisolid System of an Antihypertensive Drug

**DOI:** 10.1155/2015/574247

**Published:** 2015-10-22

**Authors:** Nilesh P. Kala, Divyesh H. Shastri, Pragna K. Shelat

**Affiliations:** Department of Pharmaceutics & Pharmaceutical Technology, K. B. Institute of Pharmaceutical Education & Research, Sector 23, Gandhinagar, Gujarat 382023, India

## Abstract

Nifedipine is an antihypertensive BCS class II drug which has poor bioavailability when given orally. The objective of the present study was to increase the bioavailability of nifedipine, by formulation and evaluation of a buccoadhesive liquisolid system using magnesium aluminium silicate (Neusilin) as both carrier and coating material and dissolution media were selected based on the solubility studies. A mixture of carboxymethylcellulose sodium and carbomer was used as mucoadhesive polymers. Buccoadhesive tablets were prepared by direct compression. FTIR studies confirmed no interaction between drug and excipients. XRD studies indicated change/reduction in crystallinity of drug. The powder characteristics were evaluated by different flow parameters to comply with pharmacopoeial specifications. The dissolution studies for liquisolid compacts and tablet formulations were carried out and it was found that nifedipine liquisolid tablets formulated from bioadhesive polymers containing 49% liquisolid system, 17.5% carbomer, and 7.5% carboxymethylcellulose sodium showed the best results in terms of dissolution properties. Prepared formulation batches were evaluated for swelling, bioadhesion strength, *ex vivo* residence time, and permeability studies. The optimized batch was showing promising features of the system. Formulating nifedipine as a buccoadhesive tablet allows reduction in dose and offers better control over the plasma levels.

## 1. Introduction

Amongst various techniques available for solubility enhancement of poorly soluble drugs, powdered liquid technology is comparatively less explored. A much advanced variant or type of powdered liquid is the liquisolid system [1]. Liquisolid technology, as described by Spireas and Bolton [1], can be used to transform a liquid into a free flowing, easily compressible, and apparently dry powder by simple physical mixing with selected excipients named the carrier material. Liquisolid technology has been applied to improve dissolution of various poorly water soluble drugs [2–5]. Drugs with poor water solubility and high first pass metabolism are difficult to deliver effectively, because gastrointestinal route must be avoided to avoid first pass metabolism. One approach is to deliver such drugs through buccal mucosa. Although not as permeable as sublingual mucosa, the buccal mucosa can still be considered an effective route of drug delivery. Since the drug is poorly soluble, it is less likely to be released effectively at the buccal mucosal membrane. In such case, a solubility enhancing technique such as the liquisolid approach can be helpful to improve absorption efficiency.

In this study, a BCS class II drug was formulated as a buccoadhesive tablet, utilizing a liquisolid approach to achieve modified release. Although sustained release formulations of nifedipine are available on the market, they were easily succeeded by new generations of antihypertensive agents which provide better control over the condition. Formulating nifedipine as a buccoadhesive tablet allows reduction in its dose and offers better control over the plasma levels. Liquisolid system containing the drug was incorporated into a matrix containing buccoadhesive polymers to formulate a buccoadhesive tablet.

## 2. Materials and Methods

### 2.1. Materials

Nifedipine (Amneal Pharmaceuticals, Ahmedabad), polyethylene glycol 400 (PEG 400) and glycerine (Sulab Reagent, Baroda), polysorbate 80 (Tween 80), carboxymethylcellulose sodium, propylene glycol and magnesium stearate (Burgoyne Burbidges & Co., Mumbai), microcrystalline cellulose (Avicel PH 102, 112, Bombay Tablets, Gandhinagar), magnesium aluminium silicate (Neusilin, Gangwal Chemicals Pvt. Ltd. Mumbai), carbomer (Acrypol 934P, Corel Pharma, Ahmedabad, India), and chitosan (Mahtani Chitosan, Veraval, India) were used as materials.

### 2.2. Solubility Studies

Solubility of nifedipine was determined in various nonvolatile solvents. Excess nifedipine was dispersed and stirred in different solvents for 48 h at 21 ± 1°C. Accurately weighed quantities of the filtered supernatants were further diluted with methanol and analyzed spectrophotometrically (UV-1800, Shimadzu, Japan) at 237 nm for their drug content. From these results, the solubility of nifedipine in the respective liquid vehicle was calculated [6].

### 2.3. Determination of the Flowable Liquid Retention Potential (Φ-Value) of a Powder

The experiment was designed to measure the flowable liquid retention potential ((Φ-value) for Avicel PH 101, Avicel PH 112, and Neusilin). To 10 g of carrier material (Neusilin), increasing amounts of nonvolatile solvent were added and mixed well. At each concentration of nonvolatile solvent added, the angle of repose for carrier was determined [7]. The corresponding Φ-value was calculated from the following equation:

(1) $$\begin{array}{c} \Phi \text{-}value=\frac{\text{Weight of liquid}}{\text{Weight of carrier or coat}}, \end{array}$$

where Φ is flowable liquid retention potential of a carrier material.

The Φ-value corresponding to an angle of repose of 33° represented the flowable liquid retention potential of carrier.

### 2.4. Selection of Carrier Material

Various powder materials, namely, Avicel PH 101, Avicel PH 112, and Neusilin, were screened based on their flowable liquid retention potential value. A Φ-value closer to 1 indicated that lesser amount of carrier material is required.

### 2.5. Preparation of Powder for Liquisolid System

Calculated quantities of drug and nonvolatile solvent were taken in 10 mL glass beaker and stirred using a magnetic stirrer to dissolve the drug in the solvent. The resulting mixture was incorporated into calculated quantities of carrier materials.

Mixing process was carried out in three steps as described by Spireas and Bolton [1]. During the first stage, the system was blended using blender (RQ 120, Remi Elektrotek Ltd., Mumbai) at an approximate mixing rate of 60 RPM for approximately 1 min in order to evenly distribute the liquid mixture in the powder. In the second stage, the liquid/powder admixture was evenly spread as a uniform layer on the surfaces of a mortar and left standing for approximately 5 min to allow drug solution to be absorbed by the powder particles. In the third stage, the powder was scraped off the mortar surfaces by means of spatula and then blended at a higher rate of RPM for another 30 sec. This yields the final liquisolid system.

### 2.6. Calculation of Quantities of Carrier Material for Liquisolid System Tablet

The success of liquisolid system with an acceptable flow rate and compressibility depends on liquid load factor (*L*_*o*_) and excipient ratio (*R*). The liquid load factor (*L*_*o*_) is a characteristic of amount of vehicle used in the formulation that is defined as the weight ratio of the liquid medication (*W*) and carrier. The excipient ratio (*R*) of a powder is defined as the ratio between the weights of carrier (*Q*) and coating material (*q*) present in the formulation, hence, the powder excipients ratio and liquid load factor of the formulations.

From the drug concentration, the dose of the drug, and carrier-coat ratio (*R*-value), weight of liquid medication (*W*) can be calculated. For sustained release purpose, *R*-value of 20 was used [8].

(*W*), weight of liquid medication, is to be calculated from %w/w, thereby multiplying dose with 100 and dividing with weight of solvent (30):

 
*L*_*o*_ is optimum liquid load factor.
 
*q* is quantity of coating material:
  (2) $$\begin{array}{c} \text{Weight of liquid medication }\left(\;W\;\right)=\frac{\left(Dose\times 100\right)}{\text{Drug solubility}}. \end{array}$$

Then using equation *L*_*o*_ = *W*/*Q*, quantity of carrier (*Q*) can be determined

(3) $$\begin{array}{c} Q=\frac{W}{L_{o}}. \end{array}$$

Then using equation *R* = *Q*/*q*, quantity (*q*) can be calculated:

(4) $$\begin{array}{c} q=\frac{Q}{R}. \end{array}$$

### 2.7. Selection of Bioadhesive Polymers

Three bioadhesive polymers, namely, carbomer, CMC, and chitosan, were mixed to prepare a 49% w/w liquisolid system that was compressed into tablets (Cadmach Machinery, Ahmedabad, India). Formulations for these tablets are presented in Table 1. These buccoadhesive tablets were tested for mucoadhesive strength and drug release. Based on these results, suitable bioadhesive polymer(s) was selected.

### 2.8. Optimization of Polymer Ratio

Based on above screening, two bioadhesive polymers, carbomer and CMC (mixture described in Table 2 replaced the individual 50 mg of mucoadhesive polymer found in *F*1–*F*3), were evaluated further to optimize the polymer ratio (Table 2). The remainder of the formulation was as described for formulations *F*1–*F*3.

### 2.9. Preparation of Buccoadhesive Tablets

Flat, 200 mg tablets were prepared by direct compression method using multistation rotary punch tablet compression machine. All tablets contained 49% liquisolid system, 50% of bioadhesive polymer (or ratio of polymers with different mixing ratios), and 1% of magnesium stearate (as lubricant). All liquisolid preparations were compacted into tablets using a sixteen-station rotary compression machine (Cadmach Machinery, Ahmedabad, India) using flat-faced punch with a compression force that provides acceptable tablet hardness. Composition of liquisolid compacts batches is shown in Table 1.

### 2.10. Precompression Studies and Evaluation of Liquisolid System

#### 2.10.1. Bulk Density

Sample of 25 gm weight (*M*), which was previously passed through 20 # sieve, was transferred in 100 mL graduated cylinder. The powder was levelled carefully without compacting, and the unsettled apparent volume was read. Apparent bulk density in gm/mL was calculated by the following formula:

(5) $$\begin{array}{c} \text{Bulk density}=\frac{\text{Weight of Powder}}{\text{Bulk Volume}}. \end{array}$$

#### 2.10.2. Tapped Density

Sample of 25 gm weight (*M*), which was previously passed through 20 # sieve, was transferred in 100 mL graduated cylinder. Then cylinder containing sample was tapped mechanically by raising the cylinder and allowing it to drop under its own weight using mechanical tapped density tester. Cylinder was tapped for 100 times and tapped volume was measured. Tapped density in gm/mL was calculated by the following formula:

(6) $$\begin{array}{c} \text{Tapped density}=\frac{\text{Weight of Powder}}{\text{Tapped Volume}}. \end{array}$$

#### 2.10.3. Angle of Repose

The angle of repose of powder blend was determined by fixed height funnel method. Angle of repose (*θ*) was calculated using the following equation:

(7) $$\begin{array}{c} \theta =\tan^{-1}\left(\;\frac{h}{r}\;\right). \end{array}$$

#### 2.10.4. Carr's Index

The compressibility index of the powder blend was determined by Carr's compressibility index [9]. The formula for Carr's index is as below:

(8) Carr's  index%=Tapped  density−Bulk  density×100Tapped  density.

#### 2.10.5. Hausner's Ratio

Hausner's ratio was calculated from the equation:

(9) $$\begin{array}{c} \text{Hausner's ratio}=\frac{\text{Tapped density}}{\text{Bulk density}}. \end{array}$$

### 2.11. Calculation of Fraction of the Drug Dissolved [8]

The ratio of the drug's solubility in the liquid vehicle (*C*_*L*_) to the drug concentration (*C*_*d*_) in the liquisolid system denotes the fraction (*F*_*M*_) of the dissolved, or molecularly dispersed, drug in the liquid medication of the prepared liquisolid tablets.

Therefore

(10) $$\begin{array}{c} F_{M}=\frac{C_{L}}{C_{d}}. \end{array}$$

It should be noted here that the fraction of the molecularly dispersed drug in any system cannot exceed unity and, thus, in the cases where *C*_*L*_ is greater than *C*_*d*_, the value of *F*_*M*_ should be set equal to 1.

### 2.12. X-Ray Diffraction Analysis

Liquisolid system was subjected to X-ray diffraction analysis to determine the crystalline state of nifedipine in the system. Analysis was carried out at diffraction angle range (2*θ*) of 10° to 70°.

### 2.13. Evaluation of Buccoadhesive Tablets

#### 2.13.1. Drug Excipient Compatibility Study by FTIR Spectroscopy

*Fourier Transform Infrared Spectroscopy (FTIR).* FTIR spectroscopy helps to determine any chemical interaction between drug and excipients used in formulation. The FTIR spectra for nifedipine and optimized powder mixture for liquisolid preparations were obtained using FTIR-8400S Spectrophotometer (Shimadzu, Japan). The pure drug and physical mixtures (nifedipine, carboxymethylcellulose sodium, Neusilin, carbomer, and magnesium stearate were added to physical mixture) were separately mixed with IR grade KBr to prepare KBr disks. These KBr discs were then scanned over a wave number range of 4000–400 cm^−1^ pressure.

#### 2.13.2. Drug Content Uniformity

Content uniformity of prepared tablets was assessed by crushing a single tablet and extracting the drug from the powder using 100 mL of methanol. Samples were analyzed by UV 1800 Spectrophotometer (Shimadzu, Japan) at 235 nm; dilutions were made with methanol [10].

#### 2.13.3. Swelling Study

Buccal tablets were weighed individually (*W*_1_), placed separately in petri dishes containing 4 mL of phosphate buffer (pH 6.8) solution. At regular intervals (1, 2, 3, 4, 5, 6, 7, and 8 h), the tablets were removed from the petri dishes and excess surface water was removed carefully using filter paper. The swollen tablets were then reweighed (*W*_2_), and swelling index (SI) was calculated using the following formula [9]:

(11) $$\begin{array}{c} \text{Swelling Index}=\frac{\left(W_{2}-W_{1}\right)}{W_{1}}. \end{array}$$

#### 2.13.4. *Ex Vivo* Bioadhesive Strength Measurement of Tablets

**Figure 1:**
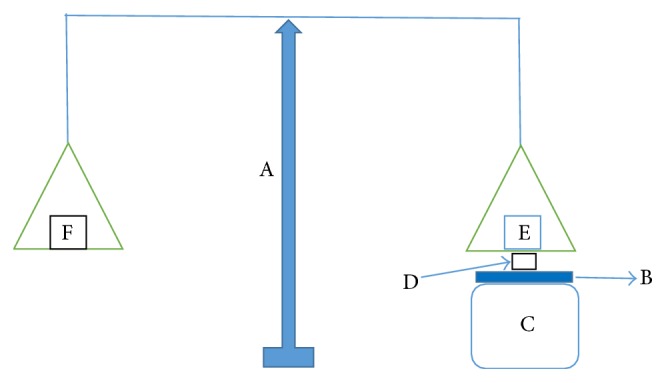
*Ex vivo* bioadhesion strength measurement.

The* ex vivo* bioadhesive strength of the prepared tablets was measured using a modified two-armed physical balance as shown in Figure 1 [11]. Freshly excised bovine buccal mucosa (obtained from a local slaughterhouse and stored in normal saline at 4°C upon collection) was used as a model tissue (E^2^). The bovine buccal mucosa (B) was fixed on the glass stage (C) using cyanoacrylate adhesive. The prepared tablet (D) was attached to the balance pan and then the glass stage (C) was raised slowly until the tablet surface came in contact with the buccal mucosa. A preload of 5 g (at E) was applied over the balance pan above the tablet for 5 min and then removed. Distilled water from a burette was added dropwise to the opposite side arm (F). Water addition was stopped at the detachment point of tablet and tissue. Amount of water required was noted from the burette and required weight was calculated

(12) $$\begin{array}{c} \text{Bioadhesion strength}=\frac{W}{\rho }, \end{array}$$

where *W* is the weight of water required for detachment and *ρ* is the density of water:

(13) Force  of  adhesion N=Bioadhesive  strength∗9.811000.

#### 2.13.5. *Ex Vivo* Residence Time Measurement of Tablets [12]

A freshly cut bovine buccal mucosa was fixed on the internal side of a beaker with cyanoacrylate adhesive. A side of each tablet was wetted with 50 *μ*L of phosphate buffer pH 6.8 and was attached to the buccal tissue by applying a light force with a fingertip for 20 s. The beaker was filled with 80 mL of phosphate buffer pH 6.8 and kept at 37 ± 1°C. After 2 min, a stirring rate of 110 RPM was applied to simulate the buccal cavity. Mucoadhesive time was monitored until complete detachment or erosion of the tablet occurred.

#### 2.13.6. *In Vitro *Release Study [13]

Dissolution studies were performed for each formulation using the USP II apparatus at 50 RPM. The dissolution medium consisted of 900 mL phosphate buffer (pH 6.8) and 0.1% w/v sodium lauryl sulphate (SLS) at 37 ± 0.58°C. At appropriate time intervals, 5 mL samples were taken and filtered through a 0.45 *μ*m Millipore filter, and the absorbance of each sample was measured spectrophotometrically at 235 nm. Periodically samples were withdrawn and same volume of fresh medium was replaced.

#### 2.13.7. *Ex Vivo* Permeability Study [14]

*Ex vivo* permeation study through the buccal mucosa was performed using a Franz diffusion cell at 37 ± 0.2°C and 50 RPM, using a magnetic stirrer. Buccal mucosa was obtained from a local slaughterhouse and used within 2 h of slaughter. The epithelium was separated from underlying connective tissues with surgical scissors and clamped between donor and receiver chambers 0.05 M^2^ of the Franz diffusion cell. After the buccal membrane was equilibrated for 30 min with phosphate buffer at pH 6.8 in both chambers, the receiver chamber was filled with fresh pH 6.8 buffer. The hydrodynamics in the receptor compartment was maintained by stirring with a magnetic bead at 50 RPM. The buccal tablet was placed in the donor chamber and 1 mL of buffer solution (pH 6.8) will be added. Aliquots (1 mL) were collected at predetermined time intervals and were replaced with the same quantity of fresh solution. The collected aliquots were filtered through a filter paper, and the amount of drug permeated through the buccal mucosa was determined by measuring the absorbance at 236 nm using a UV spectrophotometer.

Five kinetic models were used for controlled drug release curve fitting to select the most appropriate model. The dissolution data for optimized batch was fitted to the zero-order, first-order, Higuchi, Korsmeyer-Peppas, and Hixson-Crowell models. The best fit model was selected on the basis of highest correlation coefficient and lowest *F* value. Comparative statistical parameters for all the models were obtained. The results of kinetic model fitting are shown in Table 9.

## 3. Results and Discussion

### 3.1. Drug-Excipients Compatibility Study by FTIR Spectroscopy

FTIR analyses provide information on physicochemical properties of substances with respect to compatibility. FTIR and spectra of nifedipine and physical mixture of drug and optimized formulation are shown in Figure 1. IR spectrum (Figure 1) of nifedipine exhibits characteristic peaks at absorption bands in the region of 3330 cm^−1^ due to stretching vibration of N-H and aromatic –C-H stretching that appears at 3000 cm^−1^. Several bands in the region of 2962 cm^−1^–2872 cm^−1^ show methyl group having asymmetric and symmetric peaks, respectively. C=O stretching vibration appears at 1677 cm^−1^. –C-N stretching of pyridine appears at 1340 cm^−1^. Aryl nitro compounds are found in the region of 1527 cm^−1^. Appearance of all these peaks and absence of any new peaks in the physical mixture and liquisolid formulation indicated no chemical interaction between the drug and excipients.

**Figure 2:**
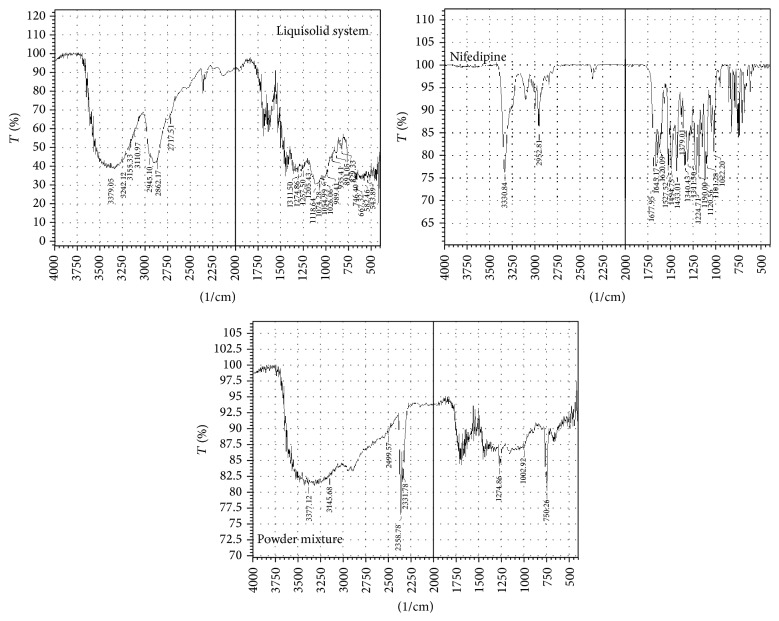
FTIR spectrum of liquisolid system, nifedipine, and precompression powder mixture.

FTIR was used for the identification of drug. The IR peaks show the presence of the functional groups in the drug molecule. Individual sample of drug, liquisolid system, and precompression powder (Figure 2) containing all excipients were analyzed by FTIR. Results indicated absence of any incompatibility between selected components.

### 3.2. XRD Study

**Figure 3:**
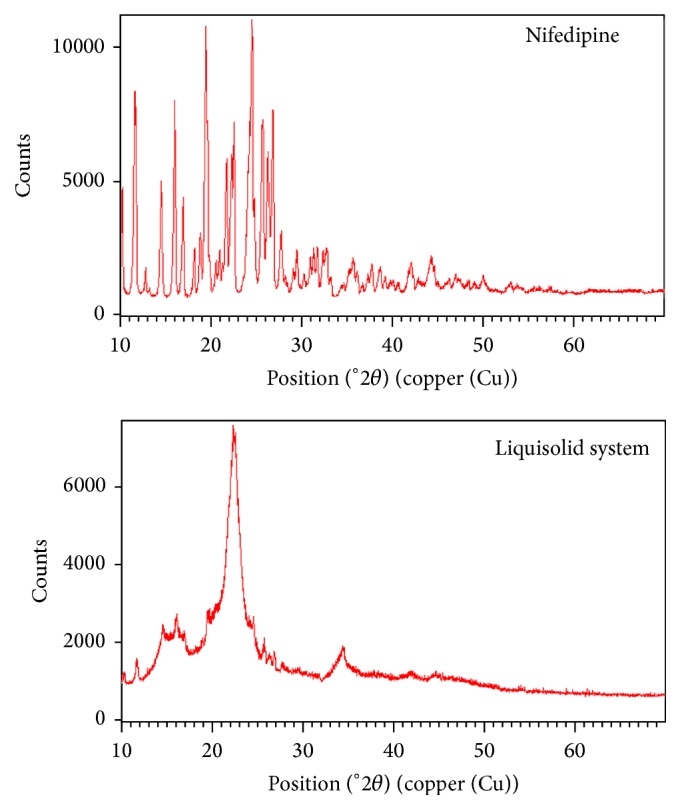
X-ray diffractogram of nifedipine and liquisolid system.

The X-ray diffraction pattern of pure nifedipine showed characteristic high intensity diffraction peaks indicating that the drug is crystalline (Figure 3) whereas reduced intensity peaks were observed for liquisolid compact (Figure 3) that might be due to the lower level of drug in the sample. This can be tested for XRD by comparison of a physical mixture of the same composition not treated to form the compact (at a diffraction angle (2*θ*) of 15°, 18°, 19°, 24°, and 26°). However, the peak at 24° can be seen clearly in liquisolid system (Figure 3). This suggests the presence of nifedipine in both crystalline and solubilized form.

### 3.3. Solubility Study of Nifedipine in Nonvolatile Vehicles

Solubility data of drug nifedipine in various liquid vehicles is shown in Table 3 which showed that the drug is more soluble in PEG 400 than other vehicles. The solubility is an important factor in liquisolid systems, as higher solubility of drug in liquid vehicle can lead to higher dissolution rates since the drug will be more molecularly dispersed and more surface of drug will be exposed to the dissolution media. PEG 400 was chosen as the nonvolatile solvent to prepare the liquisolid system.

### 3.4. Liquid Retention Potential

Measuring angle of repose is an important step in the formulation of liquisolid tablets. The relationships of angle of repose with corresponding Φ-value Avicel PH 102, Avicel PH 112, and Neusilin are shown in Table 4. The Φ-values for liquid vehicles were used to calculate *L*_*f*_. *L*_*f*_ was then used to decide the optimum amount of carrier and coating materials required to ensure dry, free flowing, and compactible powdered systems. The lowest liquid factor was obtained for Avicel PH 102 and accordingly the amount of carrier was higher than other formulations. The highest liquid factor was obtained for Neusilin and Avicel PH 112 and accordingly the amount of carrier was lower than other formulations. Neusilin has high capacity to retain liquid and was used as carrier material.

### 3.5. Calculation of Quantities of Carrier and Coat Material for Liquisolid System

*L*_*o*_ for selected carrier-coat system (Avicel PH 112 and Neusilin) can be calculated as follows:

 Φ*L*_*f*_ = Φ + Φ(1/*R*).
 Φ*L*_*f*_ = 0.75 + 0.7 (1/20).
 Φ*L*_*f*_ = 0.78 = *L*_*o*_.
 Drug solubility in selected solvent PEG 400 = 30% w/w.
 Dose of nifedipine = 10 mg.
 
*R* value = 20 [8].
 Therefore, weight of medication *W* = (dose × 100)/concentration.
 
*W* = (10 × 100)/30 = 33.33 mg.
 The quantity of carrier material can be calculated as
 
*Q* = *W*/*L*_*o*_ = 33.33/0.78 = 42.74 mg.
 The quantity of coating material can be calculated as
 
*Q* ^1^ = *Q*/*R* = 42.74/20 = 2.13 mg.

### 3.6. Characterization of Liquisolid System (Powdered Admixture)

Powder flowability is crucial in the industrial production of tablet dosage forms, as a uniform powder stream through hopper confirms uniformity of both tablet weight and drug content.

The results of various flow parameters are shown in Table 5.

The liquisolid system containing Neusilin showed good flowability as shown in Table 5. Carr's index, Hauser's Ratio, and angle of repose were found from 11 to 15, 1.12 to 1.18, and 20 to 30, respectively.

### 3.7. Fraction of Drug Dissolved

Consider

(14) FM=CLCd,FM=3.3045000,FM=7.33×10−5.

A low *F*_*M*_ value indicates that drug is largely present in undissolved form, a requisite for sustained release.

### 3.8. Postcompression Evaluation of Buccoadhesive Tablets

The tablets containing liquisolid system were evaluated for friability, hardness, thickness, drug content, bioadhesive strength, and* ex vivo* permeation study, and so forth. The results are shown in Table 6. Batches *F*4, *F*5, and *F*9 showed desired residence time. All the prepared tablets complied with the pharmacopoeial standards and specifications for the weight variation and content uniformity tests. Results of hardness, friability, thickness, bioadhesive strength, and* ex vivo* residence time are represented in Table 6. Hardness test showed an average hardness of liquisolid tablets ranging from 6.0 ± 0.35 to 8.0 ± 1.24 kg/cm^2^. Another measure of tablets strength is friability. Conventional compressed tablets that lose less than 1% w/w of their weight are generally considered acceptable. The percentage friability for all formulations was below 1% w/w, indicating that the friability is within the prescribed limits. Bioadhesive strengths of all the formulations are found within the range of 30 ± 1.5 g to 39 ± 1.5 g that indicated good bioadhesive strength. Moreover,* ex vivo* residence time found to be 4.0 ± 0.2 h to 8 ± 0.4 h was due to good bioadhesive strength and hardness of the formulations. The higher the bioadhesive strength is, the higher the residence time will be.

### 3.9. Swelling Study

Initial swelling was high in all batches. However, swelling rate slowed down after 2 h. Results are shown in Table 7. Batches *F*2 and *F*4 showed least swelling.

### 3.10. *In Vitro* Drug Release Study

**Figure 4:**
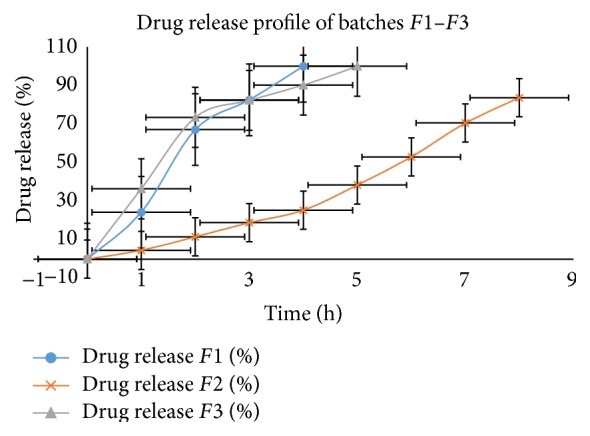
*In vitro* release profiles of batches *F*1–*F*3.

*In Vitro Drug Release of the Prepared Formulation Batches*. Batch *F*1 containing only carbomer showed complete release at 4 hrs. Batch *F*3 containing only chitosan showed complete release at 5 hrs. Batch *F*2 containing only CMC showed incomplete release even at 8 hrs (Figure 4). Based on these results, various ratios of CMC and carbomer mixture were tried.

**Figure 5:**
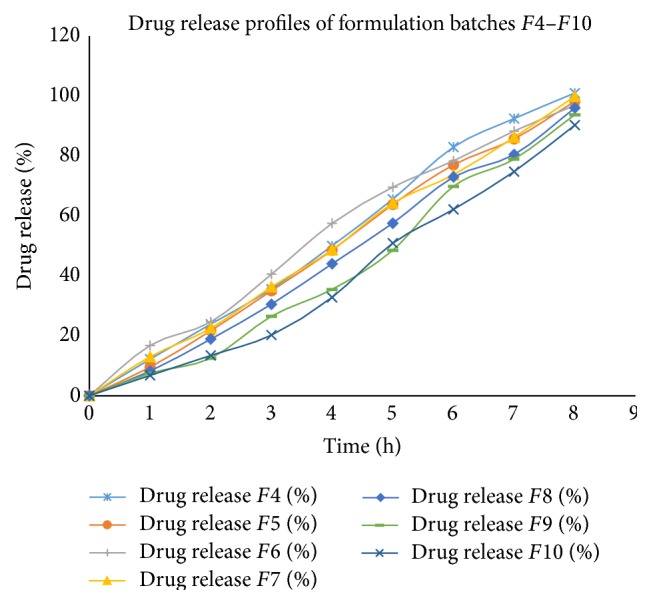
*In vitro* drug release profile of batches *F*4–*F*10.

Drug release was relatively linear in all formulations (Figure 5). Batch *F*5 showed 98.18% drug release. Batch *F*5 was subsequently selected for permeation study because it showed higher bioadhesive strength and sufficient* ex vivo* residence time.

### 3.11. *Ex Vivo* Permeation Study of Optimized Batch (*F*5)

**Figure 6:**
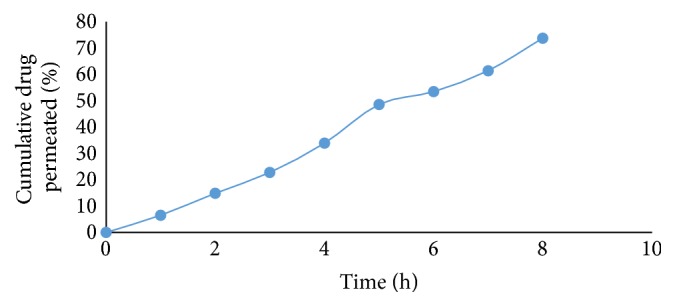
*Ex vivo* permeation study of optimized batch (*F*5).

Permeation study was performed using goat buccal mucosa. Results are shown in Table 8. A graphical representation is shown in Figure 6.

Flux (*J*_ss_) and permeability coefficients (Pc) were calculated on the basis of drug permeation

(15) Flux  Jss=3.89 μg cm−2 h−1,Pc=0.3 cm/h.

### 3.12. Release Mechanism and Kinetic Model Fitting for Optimized Batch (*F*5)

Model fitting results of the drug release data of the optimized batch revealed that Korsmeyer-Peppas model was best fitted model as *R* is 0.9981, *n* (diffusion exponent) is 1.1732 which is >0.85 indicating dissolution controlled release mechanism, and kinetic constant is 0.065.

The new technique of liquisolid system appears to be a promising alternative for the formulation of water-insoluble drugs. The higher dissolution rate displayed by liquisolid system is due to the increased wetting properties and surface of the drug available for dissolution. Estimation of fraction of drug dissolved and XRD studies suggest that only a fraction of the drug is present in dissolved form. Nifedipine is compatible with excipients used in formulation and stability study indicated that formulation is stable.

Among the bioadhesive polymers screened, carbomer showed excellent bioadhesion but provided poor control over drug release. Reason for this poor drug release might be the formation of nonpermeative, erodible matrix by carbomer. Addition of carboxymethylcellulose sodium in the formulation led to improvement in dissolution because of matrix formation by combination of these two bioadhesive polymers. Carboxymethylcellulose sodium allows water to enter into the matrix while still maintaining matrix structure. This creates a slowly diffusing matrix that allows controlled release of drug.

The use of liquisolid system while formulating buccoadhesive dosage form for low dose, poorly soluble drugs enables better control over release.

From the results of different batches prepared by Neusilin as carrier it was found that Neusilin proved to be the superior carrier than others. A lesser amount of Neusilin was required to adsorb the same amount of liquid vehicle compared to Avicel which lowered the weight of tablet. Avicel PH 112, when used as carrier material, gives lower unit dosage form size due to its high surface area. The flow property obtained by Neusilin was good and remains unaffected at such low amount. The flowability improvement can be attributed to the high porosity and high specific surface area of these excipients, which allows penetration of liquid into the particle pores resulting in a weight gain of individual particle accompanied by better flow properties. High flux observed with liquisolid bioadhesive tablet indicated that presence of drug in dissolved form significantly affects permeation across buccal mucosa.

## 4. Conclusion

Liquisolid system containing the drug was incorporated into a matrix containing buccoadhesive polymers to formulate a buccoadhesive tablet. PEG 400 proved to be promising liquid vehicle for formulation of liquisolid preparations. Nifedipine liquisolid tablets formulated from bioadhesive polymers containing 49% liquisolid system, 17.5% carbomer, and 7.5% carboxymethylcellulose sodium provided the best results in terms of dissolution properties. Neusilin was found more suitable as carrier materials instead of Avicel, as the liquid adsorption capacity increases manyfold and thus tablet weights are reduced in case of Neusilin as compared to Avicel.

## Figures and Tables

**Table 1:** Formulation of batches containing various bioadhesive polymers.

Batches	Ingredients (mg/200 mg tablet)				
	Liquisolid	Carbomer	CMC	Chitosan	Mg stearate
*F*1	98	50	—	—	2
*F*2	98	—	50	—	2
*F*3	98	—	—	50	2

**Table 2:** Formulations containing various ratio of bioadhesive polymers.

Formulation	CMC (mg/tablet)	Carbomer (mg/tablet)
*F*4	10	40
*F*5	15	35
*F*6	20	30
*F*7	25	25
*F*8	30	20
*F*9	35	15
*F*10	40	10

**Table 3:** Solubility data of nifedipine in various liquid vehicles.

Solvent	Solubility (mg/mL)
PEG 400	3.30 ± 0.75
Propylene glycol	2.28 ± 0.85
Glycerine	0.16 ± 0.90
Polysorbate 80	0.18 ± 0.48

**Table 4:** Liquid retention potential of powders.

Material	Φ-value
Avicel PH 102	0.6
Avicel PH 112	0.75
Neusilin	0.7

**Table 5:** Flow properties of liquisolid system.

Parameters	Value
Bulk density (gm/mL)	0.365
Tapped density (gm/mL)	0.431
Carr's index (%)	15.31 ± 0.47
Hauser's Ratio	1.18 ± 0.42
Angle of repose	25.72 ± 0.42

**Table 6:** Postcompression evaluation of buccoadhesive tablets.

Parameter	*F*1	*F*2	*F*3	*F*4	*F*5	*F*6	*F*7	*F*8	*F*9	*F*10
Friability %	0.3 ± 0.01	0.1 ± 0.01	0.2 ± 0.01	0.1 ± 0.01	0.1 ± 0.01	0.2 ± 0.01	0.1 ± 0.01	0.1 ± 0.01	0.1 ± 0.01	0.2 ± 0.01

Hardness (kg/cm^2^)	8.0 ± 0.73	7.0 ± 0.25	7.0 ± 0.16	6.0 ± 0.35	7.0 ± 0.44	8.0 ± 0.17	7.0 ± 0.37	6.0 ± 0.45	8.0 ± 1.24	9.0 ± 0.68

Thickness (mm)	3.87 ± 0.04	4.01 ± 0.05	4.02 ± 0.06	4.02 ± 0.09	4.01 ± 0.07	4.03 ± 0.08	4.06 ± 0.04	4.03 ± 0.05	4.01 ± 0.07	4.04 ± 0.04

Drug content (%)	99.25 ± 2.21	100.07 ± 2.7	98.76 ± 1.10	110.2 ± 1.5	98.15 ± 2.14	98.87 ± 2.18	97.45 ± 2.25	99.33 ± 2.18	100.5 ± 1.10	98.63 ± 2.21

Bioadhesive strength (g)	30.0 ± 1.5	34.0 ± 1.6	39.0 ± 1.5	37.0 ± 1.3	38.0 ± 1.6	37.0 ± 0.7	35.0 ± 0.5	38.0 ± 1.4	36.0 ± 1.6	35.0 ± 1.5

*Ex vivo* residence time (h)	4.0 ± 0.2	6.0 ± 0.4	7.0 ± 0.4	8.0 ± 0.3	8.0 ± 0.4	6.0 ± 0.2	7.0 ± 0.3	7.0 ± 0.3	8.0 ± 0.4	7.0 ± 0.3

**Table 7:** Swelling indices of buccoadhesive tablets.

Time (h)	*F*1	*F*2	*F*3	*F*4	*F*5	*F*6	*F*7	*F*8	*F*9	*F*10
2	0.16	0.19	0.14	0.18	0.15	0.2	0.23	0.27	0.3	0.15
4	0.03	0.06	0.04	0.09	0.08	0.09	0.1	0.14	0.21	0.03
6	0.05	0.02	0.08	0.05	0.05	0.06	0.07	0.09	0.12	0.06
8	0.04	0.03	0.05	0.03	0.04	0.05	0.04	0.06	0.08	0.04

**Table 8:** *Ex vivo* permeation study of optimized batch (*F*5).

Time (h)	% cumulative drug permeation
**0**	**0**
1	6.54 ± 1.10
2	14.87 ± 2.21
3	22.78 ± 1.15
4	33.89 ± 2.45
5	48.63 ± 1.15
6	53.48 ± 2.18
7	61.45 ± 1.15
8	73.83 ± 2.16

**Table 9:** Kinetic model data of optimized batch (*F*5).

Model	*R* ^2^ value	Slope	Intercept	*F* value
Higuchi	0.9860	37.2398	−36.470	3.7879
Zero	0.9961	9.6520	−4.0003	6766.2
Korse	0.9981	1.1732	−1.1819	1.2572
Hixon	0.9961	−3.217	34.6667	143.832
First	0.9517	0.1395	0.8656	943.689

## References

[1] Spireas S., Bolton S. M. Liquisolid systems and methods of preparing same.

[2] Elkordy A. A., Bhangale U., Murle N., Zarara M. F. (2013). Combination of lactose (as a carrier) with Cremophor EL (as a liquid vehicle) to enhance dissolution of griseofulvin. *Powder Technology*.

[3] Tiong N., Elkordy A. A. (2009). Effects of liquisolid formulations on dissolution of naproxen. *European Journal of Pharmaceutics and Biopharmaceutics*.

[4] Javadzadeh Y., Siahi-Shadbad M. R., Barzegar-Jalali M., Nokhodchi A. (2005). Enhancement of dissolution rate of piroxicam using liquisolid compacts. *Farmaco*.

[5] Fahmy R. H., Kassem M. A. (2008). Enhancement of famotidine dissolution rate through liquisolid tablets formulation: in vitro and in vivo evaluation. *European Journal of Pharmaceutics and Biopharmaceutics*.

[6] Hentzschel C. M., Alnaief M., Smirnova I., Sakmann A., Leopold C. S. (2012). Enhancement of griseofulvin release from liquisolid compacts. *European Journal of Pharmaceutics and Biopharmaceutics*.

[7] Javadzadeh Y., Jafari-Navimipour B., Nokhodchi A. (2007). Liquisolid technique for dissolution rate enhancement of a high dose water-insoluble drug (carbamazepine). *International Journal of Pharmaceutics*.

[8] Spireas S., Sadu S. (1998). Enhancement of prednisolone dissolution properties using liquisolid compacts. *International Journal of Pharmaceutics*.

[9] Desai K. G., Kumar T. M. (2004). Preparation and evaluation of a novel buccal adhesive system. *AAPS PharmSciTech*.

[10] Guyot M., Fawaz F. (1998). Nifedipine loaded-polymeric microspheres: preparation and physical characteristics. *International Journal of Pharmaceutics*.

[11] Kast C. E., Bernkop-Schnürch A. (2001). Thiolated polymers—thiomers: development and in vitro evaluation of chitosan-thioglycolic acid conjugates. *Biomaterials*.

[12] Mohammed F. A., Khedr H. (2003). Preparation and in vitro/in vivo evaluation of the buccal bioadhesive properties of slow-release tablets containing miconazole nitrate. *Drug Development and Industrial Pharmacy*.

[13] Varshosaz J., Dehghan Z. (2002). Development and characterization of buccoadhesive nifedipine tablets. *European Journal of Pharmaceutics and Biopharmaceutics*.

[14] Madgulkar A., Kadam S., Pokharkar V. (2009). Development of buccal adhesive tablet with prolonged antifungal activity: optimization and ex vivo deposition studies. *Indian Journal of Pharmaceutical Sciences*.

